# Enhanced Anti-Tumor Effects of Natural Killer Cell-Derived Exosomes Through Doxorubicin Delivery to Hepatocellular Carcinoma Cells: Cytotoxicity and Apoptosis Study

**DOI:** 10.3390/ijms26052234

**Published:** 2025-03-01

**Authors:** You Hee Choi, Ho Yong Kim, Jong-Oh Park, Eunpyo Choi

**Affiliations:** 1 Korea Institute of Medical Microrobotics, 43-26 Cheomdangwagi-ro, Buk-gu, Gwangju 61011, Republic of Korea; hodragon@dankook.ac.kr (H.Y.K.); jop@kimiro.re.kr (J.-O.P.); 2 Department of Mechanical Engineering, Sogang University, 35, Baekbeom-ro, Mapo-gu, Seoul 04107, Republic of Korea

**Keywords:** natural killer cells, exosomes, doxorubicin, hepatocellular carcinoma, liver cancer

## Abstract

Exosomes are nanosized extracellular vesicles secreted by various cells, including natural killer (NK) cells, and are known for their low toxicity, high permeability, biocompatibility, and strong targeting ability. NK cell-derived exosomes (NK-exos) contain cytotoxic proteins that enhance tumor-targeting efficiency, making them suitable for treating solid tumors such as hepatocellular carcinoma (HCC). Despite their potential in drug delivery, the mechanisms of drug-loaded NK-exos, particularly those loaded with doxorubicin (NK-exos-Dox), remain unclear in HCC. This study explored the anti-tumor effects of NK-exos-Dox against Hep3B cells in vitro. NK-exos-Dox expressed exosome markers (CD9 and CD63) and cytotoxic proteins (granzyme B and perforin) and measured 170–220 nm in size. Compared to NK-exos, NK-exos-Dox enhanced cytotoxicity and apoptosis in Hep3B cells by upregulating pro-apoptotic proteins (Bax, cytochrome c, cleaved caspase 3, and cleaved PARP) and inhibiting the anti-apoptotic protein (Bcl-2). These findings suggest that NK-exos-Dox significantly boost anti-tumor effects by activating specific cytotoxic molecules, offering promising therapeutic opportunities for solid tumor treatment, including HCC.

## 1. Introduction

Hepatocellular carcinoma (HCC) accounts for about 90% of all primary liver cancers, ranking sixth in cancer diagnoses and third in cancer-related deaths, and predominantly occurs in patients with underlying chronic liver disease and cirrhosis [1,2]. The development of HCC is multifactorial, involving various factors such as chronic inflammation from non-alcoholic steatohepatitis, alcohol use, and viral infections. Moreover, it is a multistep process, potentially originating from chronic liver injury and inflammation, leading to subsequent fibrosis and/or cirrhosis, and ultimately culminating in HCC [3]. Currently, the diagnosis and management of HCC are challenged by obstacles in early diagnosis and less than optimal treatment outcomes. Additionally, chemotherapy for advanced HCC has limited response rates and provides a marginal survival benefit [4]. However, the precise molecular mechanisms underlying the initiation and progression of HCC remain incompletely understood. Therefore, novel therapeutic strategies based on cancer immunotherapy are urgently needed for HCC.

Natural killer (NK) cells are key innate immune effectors that regulate target cell functions through two major cytotoxic pathways [5]. The first pathway is an intrinsic apoptosis pathway, where NK cells release cytotoxic granules (e.g., granzyme B, perforin, chemokines, and cytokines) in the immune system [6]. Perforin forms pores in the target cell membrane, allowing granzyme B to enter and trigger cell death by releasing cytochrome c [7]. The second pathway is an extrinsic apoptosis pathway, where NK cells bind death ligands (e.g., Fas ligand (FasL) and tumor necrosis factor (TNF)-related apoptosis-inducing ligand (TRAIL)) on the target cell surface, activating their cognate receptors (Fas and TRAIL-R1/TRAIL-R2) and inducing caspase-dependent apoptosis [8]. Perforin and granzyme B are also essential for NK cell-mediated tumor killing in the intrinsic pathway [9]. Additionally, the action of death ligands (e.g., FasL and TRAIL) requires transient NK cell/target conjugations to activate caspase signaling effectors (e.g., caspase 3, 7, 8, 9) and poly[ADP-ribose] polymerase (PARP). However, NK cell-based immunotherapy is less effective in treating solid tumors, including HCC, due to insufficient NK cell infiltration in the dynamic and heterogeneous tumor microenvironment (TME) [10]. Thus, novel and innovative therapeutic strategies are needed to enhance the anti-tumor effects of NK cells in cancer therapy.

Recently, exosomes, which range in size from 30 to 150 nm, have garnered significant attention due to their critical functions, including proliferation and apoptosis in intercellular communication and their ability to naturally carry mRNA, nucleic acids, lipids, and proteins [11,12]. Notably, exosomes are highly enriched in specific proteins, such as CD9, CD63, CD81, and Alix, which serve as exosomal markers across diverse cell types, including immune cells [11]. Researchers have explored the clinical potential of exosomes as biomarkers, therapeutic carriers, and drug delivery systems [13,14]. Therefore, understanding the characteristics of exosomes is a critical indicator for future clinical applications in cancer therapy. Exosomes derived from various cells contain unique proteins that perform diverse functions depending on their cellular origins [13]. Among these, NK cell-derived exosomes (NK-exos) stand out for their strong therapeutic effects and active targeting ability due to their easy diffusion and infiltration into tumor tissues. NK-exos typically contain cytotoxic proteins (e.g., granzyme B, perforin, FasL, and TRAIL), originating from NK cells, which exert potent tumor-killing effects against various cancers (e.g., melanoma, breast cancer, neuroblastoma, lung cancer, pancreatic cancer, and liver cancer) [15,16,17,18,19,20].

In our previous study, we identified that NK-exos regulate HCC targeting and cell death through two mechanisms: membrane fusion (perforin and granzyme B) and ligand–receptor interaction (FasL and TRAIL), both in vitro and in vivo. First, the perforin secreted from NK-exos forms pores in HCC cell membranes, allowing granzyme B to enter, triggering cytochrome c release from the mitochondria, and subsequently activating caspase 9. Second, FasL and TRAIL on NK-exos bind to death receptors on HCC cells, activating initiator caspase 8. These pathways converge to activate caspase 3 or 7, ultimately inducing apoptosis via PARP activation. NK-exos cytotoxicity induces caspase-dependent apoptosis. We evaluated the cytotoxicity and cell viability of NK-exos at various concentrations and time points in HCC cells (e.g., Hep3B, HepG2, and Huh7). Interestingly, NK-exos induced the highest cytotoxicity and cell viability effects in Hep3B cells [20].

Therefore, to conduct our current study, we chose Hep3B from the HCC cell lines and derived the findings from this research. By overcoming potential immunosuppression in the tumor microenvironment (TME), NK-exos offer advantages beyond NK cells alone [21,22].

Furthermore, exosomes have been developed as promising carriers for targeted drug delivery due to their specificity, safety, and stability [23]. Exosomes efficiently deliver various cargo to target cells, including therapeutic drugs for cancer treatment [24,25,26]. Notably, NK-exos enhance the anti-tumor effects of solid cancers (e.g., ovarian and breast cancer) by delivering chemotherapeutic drugs, such as cisplatin (DDP) and paclitaxel (PTX), loaded via electroporation (NK-exos-DDP and NK-exos-PTX) [27,28]. However, the characteristics and functions of NK-exos loaded with doxorubicin (Dox) (NK-exos-Dox) have yet to be studied in HCC as shown in Table S1.

Initially, this study investigated the therapeutic mechanisms and functions involved in administering NK-exos-Dox in HCC therapy, including cytotoxicity and apoptosis. Our findings indicate that NK-exos-Dox delivery can enhance potent anti-tumor effects, particularly through inducing apoptosis in Hep3B cells. Compared to NK-exos alone and free Dox, NK-exos-Dox upregulated pro-apoptotic proteins (e.g., granzyme B, perforin, Bax, cytochrome c, cleaved caspase 3, and cleaved PARP) while downregulating the anti-apoptotic protein Bcl-2. Overall, we suggest that NK-exos-based drug delivery, specifically with loaded Dox, holds promise as a novel therapeutic approach for HCC cancer chemotherapy.

## 2. Results

### 2.1. Characterization of NK-exos-Dox

**Figure 1:**
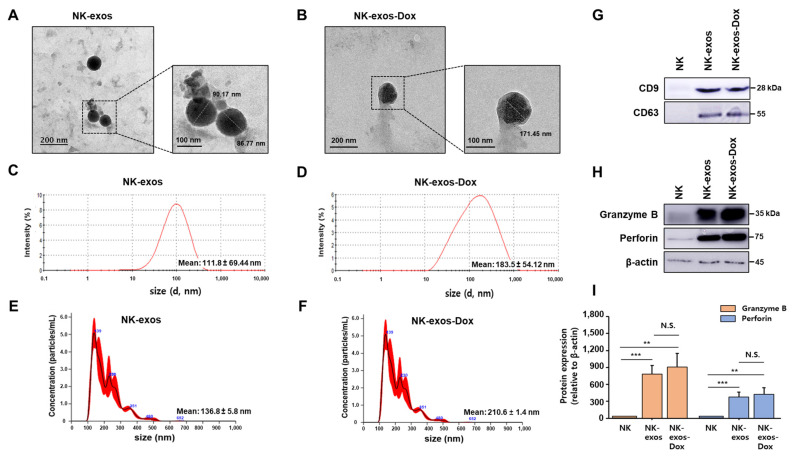
Isolation and characterization of NK-exos-Dox. (**A**,**B**) The morphologies of 50 µg of isolated NK-exos (**A**) and NK-exos-Dox (**B**) were visualized by TEM. Scale bars: 200 (left) and 100 nm (right). (**C**–**F**) Size distributions of NK-exos (**C**,**E**) and NK-exos-Dox (**D**,**F**) were analyzed using DLS (**C**,**D**) and NTA (**E**,**F**). (**G**,**H**) The expression levels of exosomal markers, including CD9 and CD63 (**G**), and cytotoxic proteins, including granzyme B and perforin (**H**) via Western blotting. β-actin was used as a loading control. Uncropped Western blot images are shown in Figures S2 and S3. (**I**) Quantification of protein expression normalized to β-actin. All data are presented as the mean ± SD (*n* = 3). ** *p* < 0.01 and *** *p* < 0.001 vs. NK cells. N.S. (no significance) vs. NK-exos.

To characterize natural killer cell-derived exosomes loaded with doxorubicin (NK-exos-Dox), we examined isolated exosomes using transmission electron microscopy (TEM), nanoparticle tracking analysis (NTA), dynamic light scattering (DLS), and Western blot analysis (WB). Exosomes are extracellular vesicles with a nano-sized range of 30–150 nm [11,29]. We confirmed the morphology of NK-exos and NK-exos-Dox using TEM (Figure 1A,B). After drug loading, there were no significant changes in the morphology of NK-exos-Dox compared to NK-exos (Figure 1B). Additionally, immunogold TEM using the CD9 exosomal marker antibody in sizes of 60 to 180 nm revealed black dots indicating the location of immunogold particles on the exosome surface, confirming positive labeling for the CD9 marker (Figure S1). Isolated NK-exos and NK-exos-Dox were analyzed for size distribution and peak diameter using DLS (NK-exos 111.8 ± 69.44 nm vs. NK-exos-Dox 183.5 ± 54.12 nm; Figure 1C,D) and NTA (NK-exos 136.8 ± 5.8 nm vs. NK-exos-Dox 210.6 ± 1.4 nm; Figure 1E,F). NK-exos-Dox exhibited peak diameters exceeding 150 nm, consistent with those previously reported for typical NK-exos [15,16,17,18,19,20,28,30,31,32]. Notably, the NK-exos-Dox particle size increased slightly (Figure 1D,F), consistent with previously reported findings [28,33,34].

To further investigate stability according to surface charge, we measured the zeta potential of NK-exos and NK-exos-Dox. The measured surface charge of NK-exos was −15.07 ± 2.88 mV; however, it significantly increased after drug loading, with NK-exos-Dox measuring −27.90 ± 0.57 mV, as shown in Table 1. Our results demonstrate a remarkable change in surface charges after electrophoresis. The increased zeta potential suggests that NK-exos-Dox may be more stable than NK-exos. Exosomes display surface protein markers that can be used for quantification and detection. Specific exosomal surface markers, including plasma membrane proteins (tetraspanins: CD9, CD63, CD81, and CD82) and multivesicular bodies (Alix and TSG101), are used to characterize exosomes [11,35]. As shown in Figure 1G and Figure S2, both NK-exos and NK-exos-Dox express these specific plasma membrane exosome protein markers (CD9 and CD63). Additionally, NK cells release exosomes that carry cytotoxic protein markers (granzyme B, perforin, FasL, and TRAIL), which play a role in inducing cell death [36]. We confirmed the expression of cytotoxic protein markers (granzyme B and perforin) via Western blot analysis (Figure 1H and Figure S3). Notably, the expression of these proteins was higher in NK-exos and NK-exos-Dox than in NK cells (Figure 1I). However, no significant difference was observed between NK-exos and NK-exos-Dox. To further validate the expression of cytotoxic proteins (perforin and granzyme B), we used mesenchymal stem cell-derived exosomes (MSC-exos) and MSC-exos loaded with Dox (MSC-exos-Dox) as negative controls. The exclusive presence of these cytotoxic proteins was confirmed only in NK-exos-Dox (Figure S4A,B). Uncropped Western blot images of perforin, granzyme B, and β-actin were analyzed using the bands marked in yellow (Figure S4C–E). Importantly, Dox loading did not significantly impact the expression of specific proteins (e.g., granzyme B and perforin) in NK-exos. Together, these results prove the successful isolation and characterization of NK-exos-Dox.

### 2.2. Loading and Release Efficiency of Dox into NK-exos as a Drug Delivery Carrier

**Figure 2:**
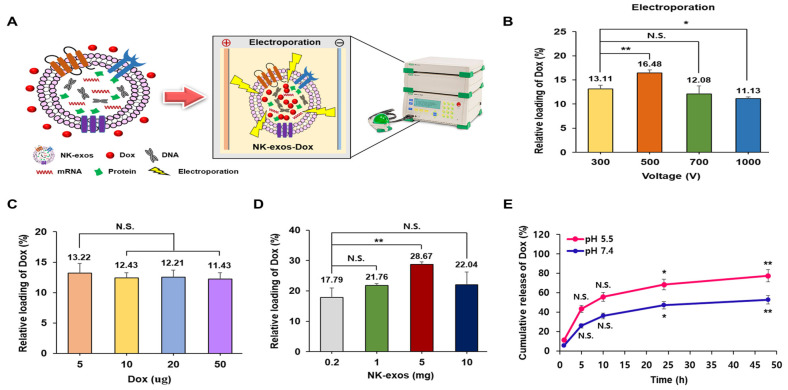
Loading and release efficiency of Dox in NK-exos using electroporation. (**A**) Schematic illustration of the preparation process for NK-exos-Dox. (**B**–**D**) The relative loading efficiency of Dox was determined by electroporation at the specified voltages (**B**), the amounts of Dox (**C**), and the amounts of NK-exos (**D**). (**E**) The release efficiency of Dox from NK-exos-Dox at pH 5.5 and pH 7.4. Data are shown as the mean ± SD (*n* = 3). * *p* < 0.05, ** *p* < 0.01, and N.S. (no significance).

This study investigated the potential of using NK-exos as delivery vehicles to carry the chemotherapeutic drug Dox for HCC treatment. First, Dox was loaded into NK-exos via electroporation, as described in the schematic diagram (Figure 2A). Subsequently, we evaluated various loading methods (e.g., electroporation, incubation, and sonication) to compare the loading efficiency of Dox into NK-exos (Figure 2B and Figure S5). The loading capacity of Dox in NK-exos was more effective via electroporation (up to 16.48%, at 500 V) compared to incubation (up to 2.25%, at 37 °C) and sonication (up to 15.39%, for 3 min). Furthermore, we assessed whether the Dox concentration affected the final Dox amount loaded into NK-exos. Interestingly, increasing the Dox concentration did not significantly affect the final quantities loaded into NK-exos. The highest Dox loading capacity was observed at 5 μg (13.22%) compared to 10 μg (12.43%), 20 μg (12.21%), and 50 μg (11.43%), as shown in Figure 2C. In addition, we determined whether the NK-exos concentration affected the final amount of Dox loaded into NK-exos. The increased NK-exos concentration had a significant effect on the final Dox amount being loaded, with the highest observed at 5 mg of NK-exos (28.67%) compared to 0.2 mg (17.79%), 1 mg (21.76%), and 10 mg (22.04%), as shown in Figure 2D. To simulate the cancer cell microenvironment and normal physiological environment, we used two different pH values (pH 5.5 and 7.4), respectively [37]. Finally, we measured the in vitro release efficiency of Dox from NK-exos-Dox at these two different pH levels. A significant burst of Dox was released during the first 5 h at pH 5.5 and 7.4, followed by a sustained release for up to 48 h (Figure 2E). The release efficiency of Dox from NK-exos-Dox was much faster in the acidic environment of pH 5.5 (up to 77.41%) than in the normal physiological environment of pH 7.4 (up to 52.71%). The faster release in an acidic environment is attributed to the decrease in the surface negative charge of NK-exos-Dox at an acidic pH, which facilitates the release of Dox from NK-exos-Dox by weakening the electrostatic attraction. The acid-dependent drug release feature of NK-exos-Dox is very beneficial for drug delivery systems in cancer therapy. These findings suggest that the loading and release efficiencies from NK-exos of the small molecule drug Dox, as a drug delivery carrier, are regulated by electroporation and in a pH-dependent manner, respectively.

### 2.3. Uptake of NK-exos-Dox by HCC Cells

**Figure 3:**
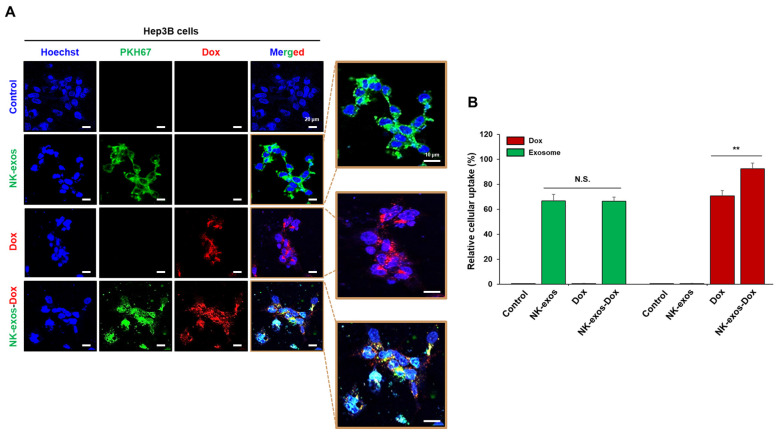
Cellular uptake of NK-exos-Dox by HCC cells. (**A**) Hep3B cells (1 × 10^4^) were co-cultured with free Dox (5 µg, red), PKH67-labeled NK-exos (50 µg, green), and PKH67-labeled NK-exos-Dox (50 µg, green + red) for 24 h. Internalization was visualized by confocal microscopy. Cell nuclei were stained with Hoechst (blue). Scale bars: 20 μm (left) and 10 μm (right). (**B**) Uptake levels of free Dox, NK-exos, and NK-exos-Dox by Hep3B cells. Data are shown as the mean ± SD (*n* = 3). N.S. (no significance) vs. NK-exos and ** *p* < 0.01 vs. free Dox.

Target cells internalize exosomes through membrane fusion, endocytosis, and receptor–ligand binding [38]. Thus, we evaluated the internalization capacity of NK-exos-Dox in Hep3B cells, an HCC cell line. The purified NK-exos were labeled with the green fluorescent dye PKH67 to assess cellular uptake. Hep3B cells were co-incubated with free Dox, PKH67-labeled NK-exos, and PKH67-labeled NK-exos-Dox for 24 h, respectively. Thereafter, green and red fluorescence were visualized using a confocal laser scanning microscope to identify the co-localization of free Dox (red), PKH67-labeled NK-exos (green), and PKH67-labeled NK-exos-Dox (red + green). All free Dox, NK-exos, and NK-exos-Dox were internalized by Hep3B cells (Figure 3A and Figure S6). The exosome uptake efficiency of NK-exos-Dox was similar to that of NK-exos alone, but the uptake efficiency of Dox in NK-exos-Dox was higher than that of free Dox (Figure 3B). NK-exos acted as a drug carrier, enhancing Dox uptake in HCC cells. These findings indicate that NK-exos-Dox are internalized and localized in Hep3B cell cytoplasm, suggesting a strong targeting effect and potential for increased anti-cancer efficacy.

### 2.4. Improvement of Cytotoxicity and Reduction in Cell Viability by NK-exos-Dox in HCC Cells

**Figure 4:**
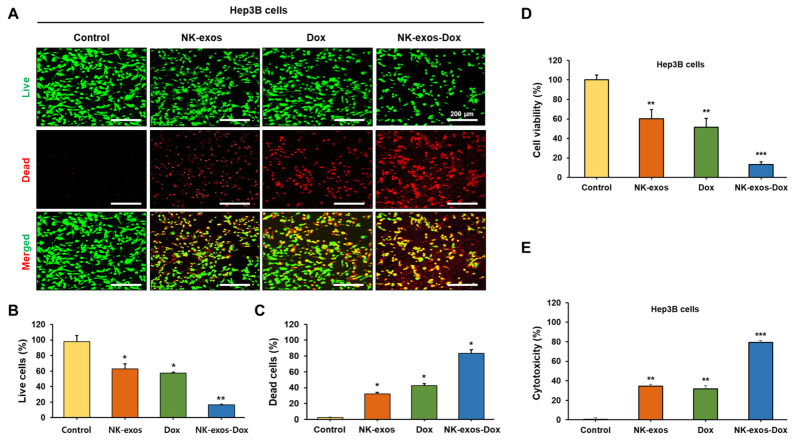
Cell viability and cytotoxicity of NK-exos-Dox in HCC cells. Hep3B cells (1 × 10^4^) were co-treated with free Dox (5 µg), NK-exos (50 µg), and NK-exos-Dox (50 µg) for 24 h. (**A**) Hep3B cells were stained using a solution containing 0.5 mM calcein-AM (green) and 4 mM EthD-1 (red) for 30 min. Live (green) and dead (red) cells were detected by live/dead assay and visualized by confocal microscopy. Scale bars: 200 μm. (**B**,**C**) Quantification of live cells (**B**) and dead cells (**C**). (**D**,**E**) Measurement of Hep3B cell viability by CCK-8 assay (**D**) and cytotoxicity by LDH assay (**E**). All data are presented as the mean ± SD (*n* = 3). * *p* < 0.05, ** *p* < 0.01, and *** *p* < 0.001 vs. untreated Hep3B cells (control).

This study investigated the biological effects of NK-exos-Dox on the cytotoxicity and cell viability of HCC cells. To assess the cytotoxic activity of NK-exos-Dox on the HCC cell line, Hep3B, we implemented a live/dead assay. Live cells were stained green, while dead cells were stained red, as visualized under a confocal microscope (Figure 4A) and quantified (Figure 4B,C). Notably, NK-exos-Dox significantly reduced the number of live cells and increased the number of dead cells (Figure 4B,C). Next, to further evaluate the anti-tumor effects of NK-exos-Dox on cell viability and cytotoxicity in Hep3B cells, we performed the CCK-8 and MTT assays for cell viability. As shown in Figure 4D and Figure S7, these findings revealed a significant reduction in cell viability after treatment with NK-exos-Dox compared to free Dox and NK-exos. Additionally, the LDH assay was used to evaluate the Hep3B cytotoxicity. Expectedly, NK-exos-Dox strongly enhanced the cytotoxicity of HCC cells (Figure 4E). Taken together, these findings suggest that NK-exos-Dox can potentially act as a drug delivery carrier to enhance cytotoxicity and reduce cell viability in HCC cells, leading to improved anti-cancer effects compared to Dox treatment alone.

### 2.5. Enhancement of Apoptotic Activity Induced by NK-exos-Dox in HCC Cells

**Figure 5:**
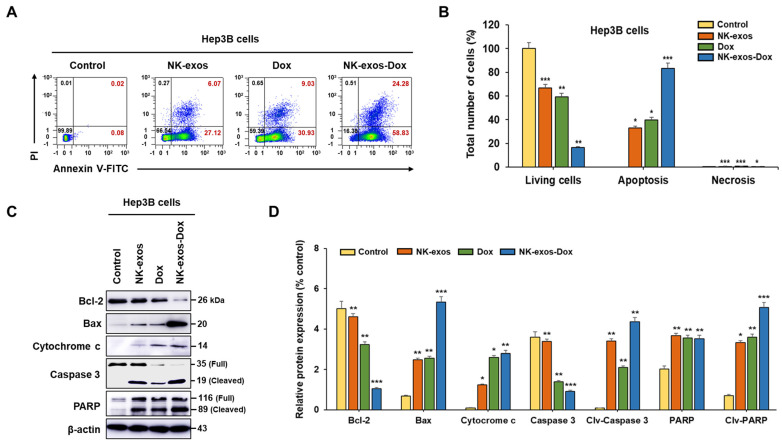
Apoptosis effects of NK-exos-Dox in HCC cells. (**A**) Hep3B cells (1 × 10^4^) were co-cultured with free Dox (5 µg), NK-exos (50 µg), and NK-exos-Dox (50 µg) for 24 h. The cells were stained with Annexin V-FITC/PI and analyzed by representative flow cytometry plots. Living cells (annexin−/ PI−); early apoptotic cells (annexin+/PI−); late apoptotic cells (annexin+/ PI+); necrotic cells (annexin−/PI+). (**B**) Quantification of living, apoptotic, and necrotic cells. (**C**) The expression levels of specific anti-apoptotic protein (Bcl-2) and pro-apoptotic proteins (Bax, cytochrome c, caspase 3, and PARP) via Western blotting. β-actin was used as a loading control. Uncropped Western blots are presented in Figure S8. (**D**) Quantification of protein expression normalized to β-actin. All data are presented as the mean ± SD (*n* = 3). * *p* < 0.05, ** *p* < 0.01, and *** *p* < 0.001 vs. untreated Hep3B cells (control).

Based on the above results of Figure 4 and Figure S7, NK-exos-Dox exhibited a significant reduction in cell viability and enhanced cytotoxicity in Hep3B cells compared to free Dox and NK-exos. Moreover, we investigated the apoptotic activity of NK-exos-Dox in HCC cells using flow cytometric analysis (Figure 5A). Exposure to NK-exos-Dox led to a significant decrease in viable Hep3B cells and a potent increase in early and late apoptotic Hep3B cells compared to free Dox and NK-exos (Figure 5B). However, exposure to NK-exos-Dox did not significantly impact necrosis (Figure 5B). These findings highlight the potent apoptotic activity of NK-exos-Dox in HCC cells.

Here, to determine the potential signaling mechanism of HCC cell apoptosis affected by NK-exos-Dox, we focused on cytotoxic proteins implicated in the intrinsic pathway (e.g., Bax, Bcl-2, cytochrome c, caspase 3, and PARP). We analyzed the expression of specific cytotoxic proteins using Western blot analysis (Figure 5C and Figure S8), which provided direct evidence of major proteins involved in apoptotic signaling. In Hep3B cells, NK-exos-Dox upregulated specific pro-apoptotic proteins, including Bax, cleaved caspase 3, and cleaved PARP, while inhibiting the anti-apoptotic protein Bcl-2, which prevents cytochrome c translocation (Figure 5D). Taken together, these findings suggest that NK-exos-Dox enhance the activation of specific cytotoxic proteins in the apoptosis signaling pathway.

### 2.6. Potential Signaling Mechanism of NK-exos-Dox Linked to Apoptosis in HCC Cells

**Figure 6:**
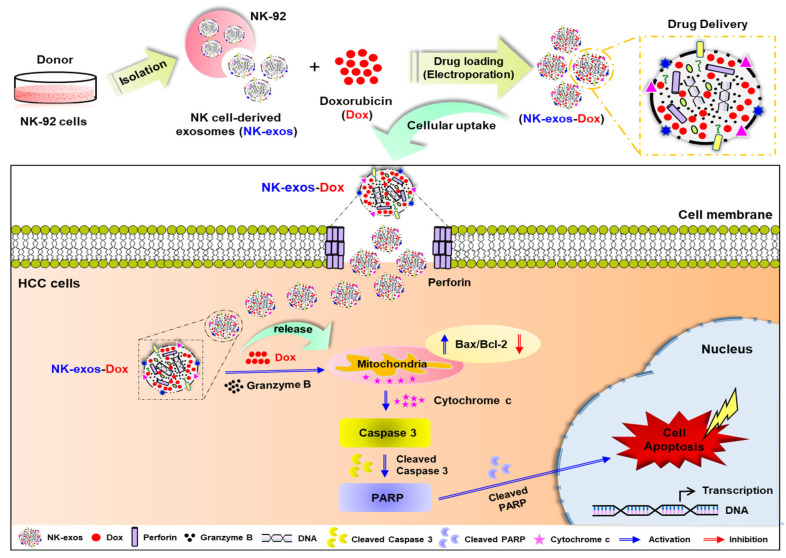
Schematic overview of NK-exos-Dox-mediated drug delivery to enhance therapeutic effects in liver cancer, specifically HCC cells. NK-exos-Dox initiate the intrinsic apoptosis pathway by transiently forming pores on the target cell membrane via perforin, releasing granzyme B and Dox into the target cells. Subsequently, they synergistically upregulate the pro-apoptotic protein (Bax) and inhibit the anti-apoptotic protein (Bcl-2), leading to the downstream activation of caspase 3 and PARP (cleaved caspase 3 and cleaved PARP). Finally, NK-exos-Dox enhance the apoptotic effect on HCC cells.

The potential signaling mechanisms through which NK-exos-Dox induce apoptosis in HCC cells are demonstrated and summarized in Figure 6. NK-exos-Dox exhibited higher levels of cytotoxic proteins, including perforin and granzyme B; however, no significant difference was noted in the presence of the chemotherapy drug Dox compared to NK-exos (Figure 1H,I). NK-exos-Dox are internalized into HCC cells before releasing cytotoxic proteins such as perforin, which form pores in the membrane. Subsequently, granzyme B and Dox synergistically initiate the mitochondrial signaling of apoptosis by interacting with the pro-apoptotic protein (Bax), inhibiting the anti-apoptotic protein (Bcl-2), and promoting caspase 3 activation (cleaved caspase 3) through cytochrome c release. Finally, NK-exos-Dox induces apoptosis in HCC cells by activating PARP (cleaved PARP). In summary, these findings provide evidence supporting the important role of NK-exos-Dox in regulating apoptosis and acting as a drug delivery system, at least in part, by enhancing the activation of key cytotoxic molecules in HCC cells.

## 3. Discussion

NK cell-based immunotherapy has revolutionized cancer treatment, showing promise for solid tumors like HCC [39], but its effectiveness in the tumor microenvironment (TME) is limited due to barriers such as restricted expansion, short lifespan, and poor penetration [2]. Meanwhile, drug delivery systems are regarded as powerful tools in cancer therapy [40]. Anti-cancer drugs are categorized into three main types: immunotherapy, hormonal therapy, and chemotherapy. In particular, chemotherapy is a widely used invasive treatment, relying on systemic distribution through oral or intravenous administration of drugs such as Dox to maximize therapeutic effects [41]. Unfortunately, its lack of selectivity often causes severe damage to rapidly dividing healthy cells, including bone marrow, hair follicles, and gastrointestinal epithelium [42]. Thus, new cancer treatment strategies are urgently required due to the limitations of NK cell therapy and chemotherapy drug delivery. Exosomes have gained significant attention in cancer therapeutics due to their unique properties, including low toxicity, high permeability, biocompatibility, and strong targeting ability [11,14,43]. They play an important role in cell proliferation and apoptosis by transporting mRNA, nucleic acids, lipids, and proteins [11,12] and also act as natural drug carriers, making them promising approaches for chemotherapy drug delivery in cancer treatment [23,26,44,45]. Notably, NK-exos exhibit strong therapeutic effects and precise targeting through cytotoxic proteins (e.g., granzyme B and perforin), effectively killing tumors in various cancers [15,16,17,18,19,20]. As shown in Table S1, a recent study showed that NK-exos enhance anti-tumor effects in solid cancers (e.g., ovarian and breast cancer) by delivering chemotherapeutic drugs such as cisplatin (DDP) and paclitaxel (PTX) via electroporation (NK-exos-DDP, NK-exos-PTX) [27,28]. However, the characteristics and functions of doxorubicin-loaded NK-exos (NK-exos-Dox) remain largely unknown in solid tumors, especially HCC.

In this study, we first demonstrated that NK-exos-Dox enhance anti-tumor activity and tumor-targeting of primary liver cancer, HCC, in vitro. Interestingly, the NK-exos-Dox delivery system was successfully developed. Dox partially incorporated into the lipid bilayer of NK-exos, causing NK-exos-Dox to slightly increase in size to approximately 220 nm after drug loading, with no morphological changes observed (Figure 1). This is consistent with the results obtained by other researchers [34,46]. Exosomes must be taken up by the target cells via several mechanisms, including endocytosis, receptor–ligand binding, and membrane fusion, to exert biological effects such as cytotoxicity and apoptosis [29,38,47]. NK-exos act as drug delivery carriers and significantly enhance the uptake of Dox in HCC cells. NK-exos-Dox have the ability to internalize more effectively into Hep3B cells compared to free Dox, suggesting a potentially high targeting effect that could improve anti-cancer efficacy (Figure 3). However, the detailed mechanism of the differential uptake of NK-exos-Dox is still unclear. Therefore, further studies are required to understand which mode of internalization contributes to the differential uptake of NK-exos-Dox in HCC cells. In our previous reports, we evaluated the internalization of NK-exos into HCC cells (e.g., Hep3B, HepG2, and Huh7), and it was shown that NK-exos were taken up in these cells. Interestingly, Hep3B cells induced the highest cytotoxicity compared with HepG2 and Huh7 cells [20]. Therefore, comparing the uptake ability of NK-exos-Dox in other HCC cells would also be meaningful for our study.

Previous studies have shown exosomes derived from dendritic cells (DCs) and NK cells can exert an immunomodulatory effect on target cells. DC-exos not only indirectly stimulate adaptive T cell responses by expressing functional membrane MHC and co-stimulatory molecules but can also directly activate NK cells by expressing functional membrane TNF superfamily ligands (TNFSFLs) such as TNF, FasL, and TRAIL [48]. NK-exos also contain cytotoxic proteins (e.g., perforin, granzyme B, TRAIL, and FasL). The expression of these proteins was found to be higher in NK-exos than in NK-92 cells [20]. NK-exos-Dox showed higher enrichment of cytotoxic proteins (e.g., granzyme B and perforin) compared to NK cells. Many recent studies have reported that NK-exos may enhance tumor-targeting and anti-tumor activity by upregulating cytotoxic proteins (e.g., perforin, granzyme B, FasL, TRAIL, and caspases) in various cancers (e.g., melanoma, neuroblastoma, breast cancer, lung cancer, and pancreatic cancer) [15,16,17,18,19].

Notably, our previous studies initially reported that NK-exos exhibit an active targeting ability and potent therapeutic effects in both orthotopic and subcutaneous HCC mouse models [20]. The functional enhancement of NK-exos by IL-15 and IL-21 synergistically upregulated cytotoxicity and apoptosis against HCC [32]. While Dox-based chemoembolization is a key therapy for HCC, its effectiveness is limited by pre-existing and acquired tumor resistance [49]. To solve these problems, NK-exos loaded with chemotherapeutic drugs can enhance the anti-tumor effects in various solid tumors through synergistic mechanisms [27,28]. However, the mechanisms of cytotoxicity and apoptosis mediated by NK-exos-Dox in HCC are still poorly understood.

Our findings revealed that NK-exos-Dox as a drug delivery system could potentially enhance the cytotoxicity and reduce cell viability against Hep3B cells, leading to better anti-cancer effects than Dox treatment alone (Figure 4). Moreover, NK-exos-Dox, as regulators of apoptosis, modulate the functions of major cytotoxic molecules by activating specific pro-apoptotic proteins (e.g., Bax, cleaved caspase 3, and cleaved PARP) while inhibiting the anti-apoptotic protein (Bcl-2), which prevents the translocation of cytochrome c (Figure 5). According to our data, NK-exos-Dox can enhance the anti-tumor activity of Hep3B cells in vitro and increase the expression of genes related to NK cell function. Therefore, NK-exos-Dox have the potential to contribute to reverse immunosuppression by recovering defective NK cells in TME. Taken together, our findings have first demonstrated the potential application and have provided novel insights into the underlying mechanisms of NK-exos-Dox in the anti-tumor therapy of HCC in vitro, as summarized in Figure 6.

Herein, we have confirmed that NK-exos-Dox may be a promising candidate for immunotherapy in solid tumors such as HCC, overcoming the limitations of NK-exos alone and free Dox. To our knowledge, this is the first report on NK-exos-based drug delivery and the enhancement of immunosuppressive capacity for HCC therapy. However, further investigations regarding NK-exos-Dox are still needed. Here, our findings are limited to the cellular level and the intrinsic apoptotic pathway (also called the mitochondrial pathway) mechanism observed in vitro. To further understand the potential role of NK-exos-Dox in HCC therapy, several steps are essential. First, investigating the anti-tumor effects of the extrinsic apoptotic pathway mediated by death ligands (e.g., FasL and TRAIL) is crucial. Second, research on the anti-tumor effects in vivo will be necessary. As shown in Table S1, NK-exos loaded with chemotherapeutic drugs (e.g., DDP and PTX) demonstrated anti-tumor effects through the activation of cytotoxic proteins-induced apoptosis pathways [27,28]. Third, further studies are needed to investigate whether NK-exos-Dox can enhance tumor-targeting and anti-tumor activity by improving cytotoxicity and apoptosis pathway-dependent cytotoxic proteins in various cancers, which could strengthen the immune response. Our study may represent a starting point for the further evaluation of NK-exos-Dox in the treatment of solid tumors.

## 4. Materials and Methods

### 4.1. Cell Lines and Cell Culture

Human NK-92 cells were purchased from American Type Culture Collection (ATCC, Manassas, VA, USA) and cultured in alpha minimum essential medium (α-MEM, Corning, NY, USA), comprising 12.5% fetal bovine serum (FBS; Corning), 12.5% horse serum (HS; Sigma-Aldrich, St. Louis, MO, USA), 1% % penicillin–streptomycin (P/S, Corning), 0.2 mM Myo-inositol (Sigma-Aldrich), 0.1 mM 2-mercaptoethanol (Sigma-Aldrich), 0.02 mM folic acid (Sigma-Aldrich), 10 ng/mL IL-2 (Miltenyi Biotec, Bergisch Gladbach, Germany), and 20 ng/mL IL-15 (Miltenyi Biotec) at 37 °C with 5% CO_2_. Hep3B cells were obtained from the Korean Cell Line Bank (KCLB, Seoul, Republic of Korea) and cultured in Dulbecco’s minimum essential medium (DMEM, Corning), supplemented with 10% FBS (Corning) and 1% P/S (Corning) at 37 °C with 5% CO_2_. Bone marrow MSCs were purchased from ATCC and cultured in α-MEM, supplemented with 10% FBS (Corning) and 1% P/S (Corning) at 37 °C with 5% CO_2_. Cells were tested for mycoplasma using an e-Myco™ Mycoplasma PCR detection kit (#25235, iNtRON Biotechnology Inc., Seongnam-si, Republic of Korea) according to the manufacturer’s protocol.

### 4.2. Exosome Isolation

NK-92 cell-derived exosomes (NK-exos) and mesenchymal stem cell-derived exosomes (MSC-exos) were isolated by multistep centrifugation, following previously described methods [50]. In brief, NK-92 cells (1 × 10^7^/well) were cultured and supplemented with exosome-depleted FBS and HS reagent, which had been prepared by ultracentrifugation (Himac CP100NX; HITACHI, Tokyo, Japan) at 120,000× *g* at 4 °C for 18 h [51], and MSCs (1 × 10^6^/well) were cultured. The culture medium underwent sequential centrifugation first at 300× *g* for 5 min, then at 2000× *g* for 15 min, and finally, the collected supernatant was centrifuged at 10,000× *g* for 30 min to remove dead cells and debris. Subsequently, the supernatant exosomes were filtered through a 0.22 μm and concentrated using a tangential flow filter system (TFF-Easy; HansaBioMed Life Sciences, Tallinn, Estonia). The exosomes were dissolved in 1 mL of phosphate-buffered saline (PBS; Corning) and either used immediately or stored at −80 °C.

### 4.3. Exosome Characterization

To evaluate morphological characteristics, we performed transmission electron microscopy (TEM) analysis on the isolated exosomes. The procedure involved fixing the exosomes onto Formvar/carbon-coated TEM grids (Agar Scientific, Stansted Mountfitchet, UK) using 2% paraformaldehyde. Next, we treated the grids with 0.05 M glycine for 10 min to quench any free aldehyde groups. Subsequently, we blocked the grids using a blocking buffer (PBS containing 1% BSA) for 30 min. Then, the exosomes were sequentially incubated with a specific CD9 antibody (EXOAB-CD9A-1; System Biosciences, Palo Alto, CA, USA) for 3 h, followed by incubation with goat anti-rabbit IgG conjugated to gold particles (Alexa Fluor 488, A-31566; Invitrogen Life Technologies, Grand Island, NY, USA) for 3 h, followed by incubation with goat anti-rabbit IgG conjugated to gold particles (Alexa Fluor 488, A-31566; Invitrogen Life Technologies, Grand Island, NY, USA) for 1 h. After washing the grids twice with a small drop of PBS, they were left to dry. Finally, we observed the exosomes on the grids using a JEM-1400 TEM (JEOL, Tokyo, Japan) operated at 80 kV, using a Mega View Camera (EMSIS, Muenster, Germany) to capture digital images.

The exosome particle sizes were determined using dynamic light scattering (DLS) with a Zetasizer Nano ZS (Malvern Instruments, Malvern, UK) equipped with a 633 nm laser. Before analysis, exosomes were placed in a square cuvette (1 mL, 1:100 dilution with PBS).

Furthermore, the exosome numbers and sizes (10 μL, 1:100 dilution with PBS) were determined by nanoparticle tracking analysis (NTA) using the Nanosight LM10 system (Malvern Instruments, Worcestershire, UK) with a 405 nm laser. These measurements were conducted using NTA software (Nanosight version 3.1; Malvern Instruments).

### 4.4. Drug Loading and Release

Exosome loading methods include drug co-incubation, electroporation, and sonication. To prepare doxorubicin (Dox; Sigma Aldrich)-loaded NK-exos (NK-exos-Dox), electroporation was conducted in 4 mm cuvettes at 500 V and 125 μF using a Gene Pulser Xcell Electroporator (Bio-Rad, Hercules. CA, USA); 50 µg of free Dox was mixed with 500 µg of NK-exos in 500 µL of electroporation buffer (600 mM sucrose) at room temperature. NK-exos-Dox were then incubated at 37 °C for 30 min to allow membrane recovery. For other methods, NK-exos and Dox were incubated at 4, 25, and 37 °C for 30, 90, and 150 min while shaking on a vortex shaker. In the sonication method, NK-exos and Dox were sonicated using a bath sonicator (Bandelin electronic GmbH & Co., KG, Berlin, Germany). Afterward, NK-exos-Dox were ultracentrifuged at 100,000× *g* for 90 min to isolate exosomes and remove unincorporated free Dox. The resulting NK-exos-Dox were resuspended in cold PBS. To quantify the encapsulated doxorubicin in NK-exos-Dox, the intrinsic Dox fluorescence was measured using a microplate reader (Varioskan Flash; Thermo Fisher Scientific, Waltham, MA, USA) with excitation and emission wavelengths of 470 nm and 590 nm, respectively, compared to the fluorescence signal against a set of known Dox standards.

To assess the release of Dox from NK-exos-Dox, 3 mL of NK-exos-Dox solution was transferred into a dialysis tube with a molecular weight cut-off of 14 kDa. The tube was first placed in a PBS buffer (10 mL, pH 7.4) and subsequently in an acetate buffer (10 mL, pH 5.0). The release of Dox occurred at 37 °C. The dialysate was collected for UV-Vis spectrophotometer analysis at the designated time intervals, and a fresh buffer solution was used as a replacement. The Dox concentrations were determined based on standard curves corresponding to the buffer solutions.

### 4.5. Exosome Uptake Assay

Exosomes were labeled with PKH67 (Green Fluorescent Cell Linker Mini Kit; Sigma-Aldrich) for green fluorescent cell labeling, according to the manufacturer’s instructions. PKH67-labeled exosomes (50 µg) and free Dox (5 µg) were incubated with Hep3B cells (1 × 10^4^/well) at 37 °C for 24 h. Aliquots of the cell suspension were placed on microscope slides and mounted with a coverslip using Aqua-Poly/Mount (Polysciences, Inc., Warrington, PA, USA). Nuclei were stained blue using Hoechst 33258 (Sigma-Aldrich). The cellular uptake of exosomes was visualized using a confocal laser scanning microscope (CLSM; Carl Zeiss, Oberkochen, Germany).

### 4.6. Live and Dead Assay

Hep3B cells (1 × 10^4^/well) co-cultured with free Dox (5 µg), NK-exos (50 µg), and NK-exos-Dox (50 µg) were measured using a live/dead assay kit (Thermo Fisher Scientific), following the manufacturer’s protocol. After 24 h, the cells were washed and incubated in PBS containing 4 μM ethidium homodimer I (red) and 0.5 μM calcein-AM (green) for 30 min. Fluorescence images were obtained using a confocal microscope (Carl Zeiss, #LSM 880), where green and red fluorescence signals indicated living and dead cells, respectively.

### 4.7. Cell Cytotoxicity Assay

Cytotoxic effects were analyzed using a lactate dehydrogenase (LDH) cytotoxicity assay kit (Thermo Fisher Scientific) following the manufacturer’s protocol. In brief, Hep3B cells (1 × 10^4^/well) were co-cultured with free Dox (5 µg), NK-exos (50 µg), and NK-exos-Dox (50 µg). Subsequently, Hep3B cells were centrifuged at 600× *g* for 5 min to separate the cellular debris from the media. Next, 50 μL of LDH reaction mixture was added to 50 μL of collected media and re-incubated in the dark at 25 °C for 30 min. The reaction was stopped by adding 50 µL of LDH stop solution. Finally, absorbance was measured at 490 nm and 680 nm using a microplate reader (Varioskan Flash; Thermo Fisher Scientific) to determine cytotoxic activity.

### 4.8. Cell Viability Assay

Cell viability effects were analyzed using a cell counting kit-8 (CCK-8; Dojindo Molecular Technologies, Kumamoto, Japan) and a 3-(4,5-dimethylthiazol-2-yl)-2,5-diphenyltetrazolium bromide (MTT; Dojindo Molecular Technologies) assay, following the manufacturer’s instructions. In brief, Hep3B cells (1 × 10^4^/well) were co-cultured with free Dox (5 µg), NK-exos (50 µg), and NK-exos-Dox (50 µg). For the CCK-8 assay, 10 μL of CCK-8 solution was added to each well and re-incubated at 37 °C for 2 h. Absorbance was then measured at 450 nm using a microplate reader (Thermo Fisher Scientific). For the MTT assay, 10 μL of MTT solution was added to each well and re-incubated for 3 h. The medium was removed, and 100 μL of dimethyl sulfoxide (DMSO) was added. Absorbance was measured at 570 nm using a microplate reader (Thermo Fisher Scientific). The cell viability was calculated relative to that of the control cells.

### 4.9. Apoptosis Analysis

The apoptotic effects were assessed using an Annexin V-FITC apoptosis detection kit (Sigma-Aldrich), following the manufacturer’s instructions. Briefly, Hep3B cells (1 × 10^6^/well) were seeded and co-cultured with free Dox (5 µg), NK-exos (50 µg), and NK-exos-Dox (50 µg). Cells were harvested and suspended in 500 μL of Annexin V-FITC-binding buffer. The mixture was subsequently analyzed using a flow cytometer (MACSQuant VYB; Miltenyi Biotec) according to the manufacturer’s protocol.

### 4.10. Western Blot Analysis

To analyze protein expression, NK-92 cells, Hep3B cells, MSCs, and isolated exosomes were lysed in RIPA buffer (GenDEPOT, Barker, TX, USA) with 1% protease inhibitor (GenDEPOT). The protein *lysates* (30 µg) of NK-92 cells, Hep3B cells, MSCs, NK-exos, and NK-exos-Dox were separated using 10% sodium dodecyl sulfate polyacrylamide gel electrophoresis (SDS-PAGE; Bio-Rad, USA). Subsequently, the gels were transferred to PVDF membranes (Millipore, Billerica, MA, USA) and blocked with 5% non-fat dry milk (Sigma-Aldrich) in 1×TBS with 0.1% Tween-20 (TBST; pH 7.4; GenDEPOT) at room temperature for 1 h. The membranes were then incubated with specific primary antibodies (diluted 1:1000) overnight at 4 °C and horseradish peroxidase-conjugated secondary antibodies (diluted 1:5000; Santa Cruz Biotechnology, Dallas, TX, USA) at room temperature for 2 h. Finally, the blots were visualized using the AmershamTM Imager 6000 imaging system (GE Healthcare, Chicago, IL, USA) and quantitatively analyzed using ImageJ software (Ver.1.53e; NIH, Bethesda, MD, USA).

The primary antibodies used were as follows: CD9 (EXOAB-CD9A-1) from System Biosciences (Palo Alto, CA, USA); CD63 (sc-5275), perforin (sc-373943), granzyme B (sc-8022), and β-actin (sc-47778) from Santa Cruz Biotechnology (Dallas, TX, USA); caspase 3 (#9662), PARP (#9532), Bax (#5023), Bcl-2 (#15071), and cytochrome c (#4280) from Cell Signaling Technology (Danvers, MA, USA).

#### Statistical Analysis

The data are presented as the mean ± standard deviation (SD) of three independent experiments and analyzed by Student’s *t*-test. Significance levels are presented as * *p* < 0.05, ** *p* < 0.01, *** *p* < 0.001, and N.S. (no significance).

## 5. Conclusions

In summary, our research findings provide the first evidence that NK-exos-Dox represents a promising therapeutic strategy for liver cancer, supported by previous studies demonstrating the superior efficacy of chemotherapeutics loaded into NK-exos compared to free drugs in vitro. Based on these results, NK-exos-Dox prepared via electroporation can offer novel insights into the therapeutic strategy for liver cancer, specifically HCC. NK-exos-Dox, as a drug carrier, enhances anti-tumor effects by upregulating cytotoxic molecules in the apoptosis pathway and efficiently promoting the cellular uptake of both NK-exos and Dox by HCC cells. Thus, as an alternative to chemotherapy, NK-exos loaded with a Dox-based drug delivery platform can provide new opportunities for liver cancer treatment and establish a strong foundation for the further evaluation of NK-exos in other solid tumor therapies.

## Figures and Tables

**Table 1:** Comparative DLS, NTA, and surface charge results of NK-exos and NK-exos-Dox. Data are shown as the mean ± SD (*n* = 3).

Sample	Size by DLS (nm)	Size by NTA (nm)	Zeta Potential (mV)
NK-exos	111.8 ± 69.44	136.8 ± 5.8	−15.07 ± 2.88
NK-exos-Dox	183.5 ± 54.12	210.6 ± 1.4	−27.90 ± 0.57

## Data Availability

The data and materials used to support the findings of this study are available from the corresponding author upon request.

## Supplementary Materials

The following supporting information can be downloaded at https://www.mdpi.com/article/10.3390/ijms26052234/s1.

## Abbreviations

The following abbreviations are used in this manuscript:

HCC	Hepatocellular carcinoma
NK-exos	NK cell-derived exosomes
NK-exos-Dox	NK-exos loaded with doxorubicin
Dox	Doxorubicin
PTX	Paclitaxel
DDP	Cisplatin
CTX	Cyclophosphamide
FasL	Fas ligand
TNF	Tumor necrosis factor
TME	Tumor microenvironment
TEM	Transmission electron microscopy
DLS	Dynamic light scattering
NTA	Nanoparticle tracking analysis
SPION	Superparamagnetic iron oxide nanoparticle
CCK-8	Cell counting kit-8
MTT	3-(4,5-dimethylthiazol-2-yl)-2,5-diphenyltetrazolium bromide
LDH	Lactate dehydrogenase
Bax	Bcl-2-associated X protein
Bcl-2	B-cell lymphoma 2
Caspase 3	Cysteine-aspartic acid protease 3
PARP	Poly (ADP-ribose) polymerase
Clv	Cleaved
