# Supplementary material for: Enhanced Anti-Tumor Effects of Natural Killer Cell-Derived Exosomes Through Doxorubicin Delivery to Hepatocellular Carcinoma Cells: Cytotoxicity and Apoptosis Study
Source: Int J Mol Sci. 2025 Mar 1;26(5):2234. doi: 10.3390/ijms26052234 (PMC11900065; doi:10.3390/ijms26052234)
Supplement: Supplementary file 1 [file ijms-26-02234-s001.zip › ijms-3443930-supplementary.pdf]

Supplementary Materials

Enhanced Anti-Tumor Effects of Natural Killer Cell-Derived Exosomes through Doxorubicin Delivery to Hepatocellular Carcinoma Cells: Cytotoxicity and Apoptosis Study

You Hee Choi[1,*], Ho Yong Kim[1], Jong-Oh Park[1] and Eunpyo Choi[2,*]

[1] Korea Institute of Medical Microrobotics, 43-26 Cheomdangwagi-ro, Buk-gu, Gwangju 61011, Republic of Korea; youheechoi@kimiro.re.kr (Y.H.C)

[2] Department of Mechanical Engineering, Sogang University, 35, Baekbeom-ro, Mapo-gu, Seoul 04107, Republic of Korea; echoi@sogang.ac.kr (E.C)

* Correspondence: youheechoi@kimiro.re.kr (Y.H.C); echoi@sogang.ac.kr (E.C)

Supplementary Figures

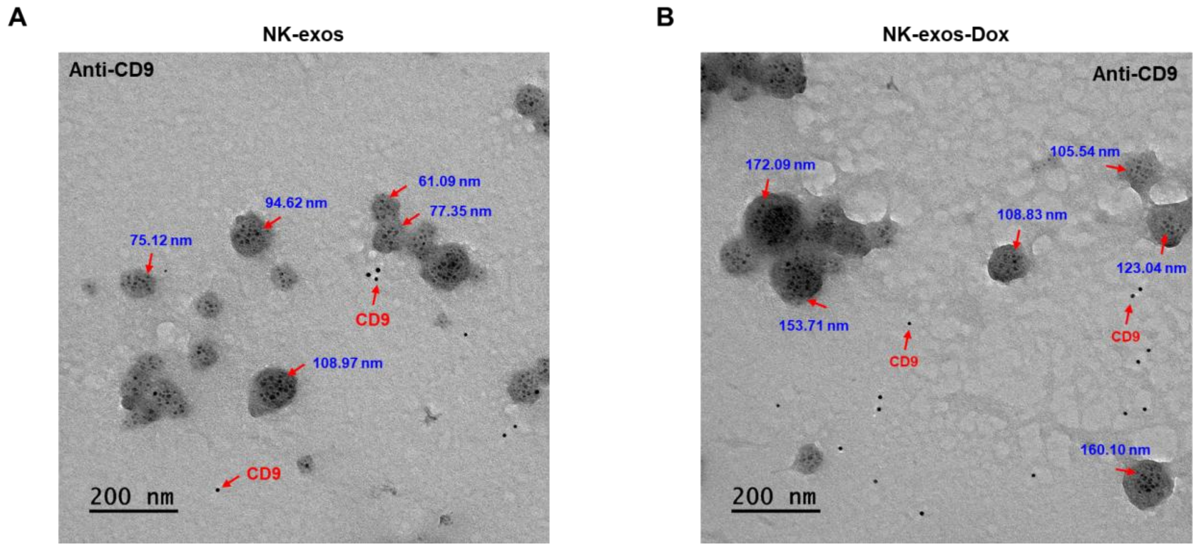

Figure S1. The morphological images of isolated NK-exos (50 μg) and NK-exos-Dox (50 μg) via TEM analysis. **(A,B)** NK-exos (A) and NK-exos-Dox (B) were visualized by immune TEM using immunogold-labling of anti-CD9 (a known marker of exosome). Gold particles are depicted as black dots and indicated by red arrows. Scale bars: 200 nm. **Abbreviations**: TEM, transmission electron microscopy.

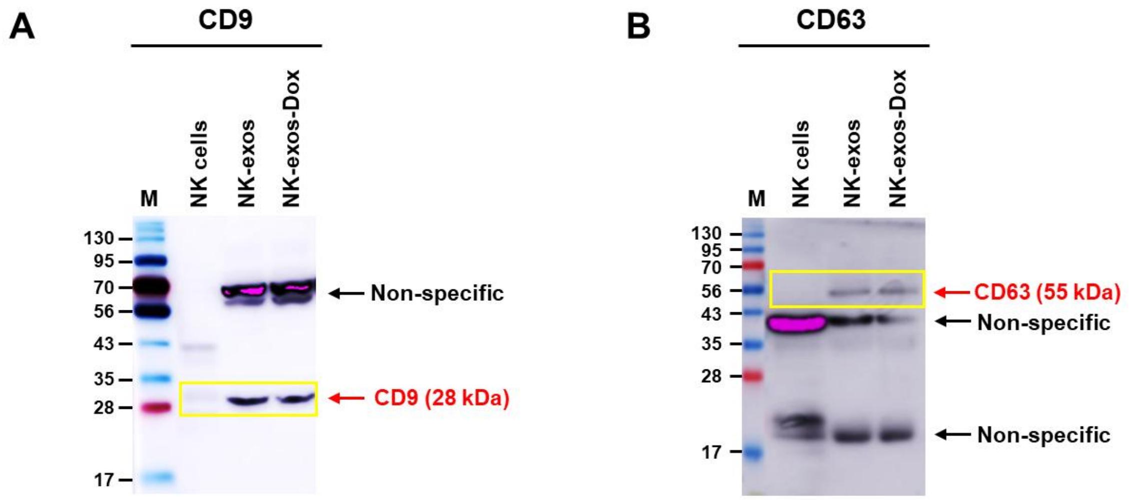

Figure S2. Uncropped western blots of exosomal markers from Figure 1G. **(A,B)** The expression levels of exosomal markers, CD9 (A) and CD63 (B). Specific expression band was indicated the yellow box and red arrow. **Abbreviations**: M, protein marker.

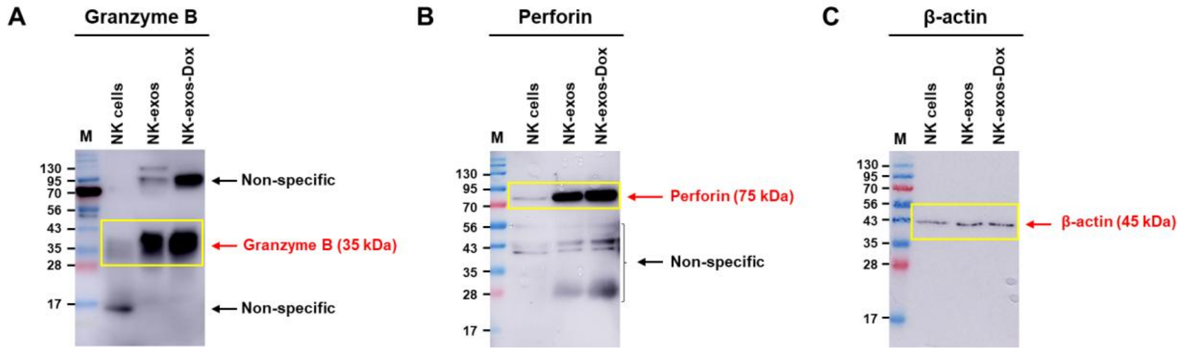

Figure S3. Uncropped western blots of cytotoxic protein markers from Figure 1H. **(A,B)** The expression levels of granzyme B (A) and perforin (B) as cytotoxic proteins. **(C)** β-actin was used as a loading control. Specific expression band was indicated the yellow box and red arrow. **Abbreviations**: M, protein marker.

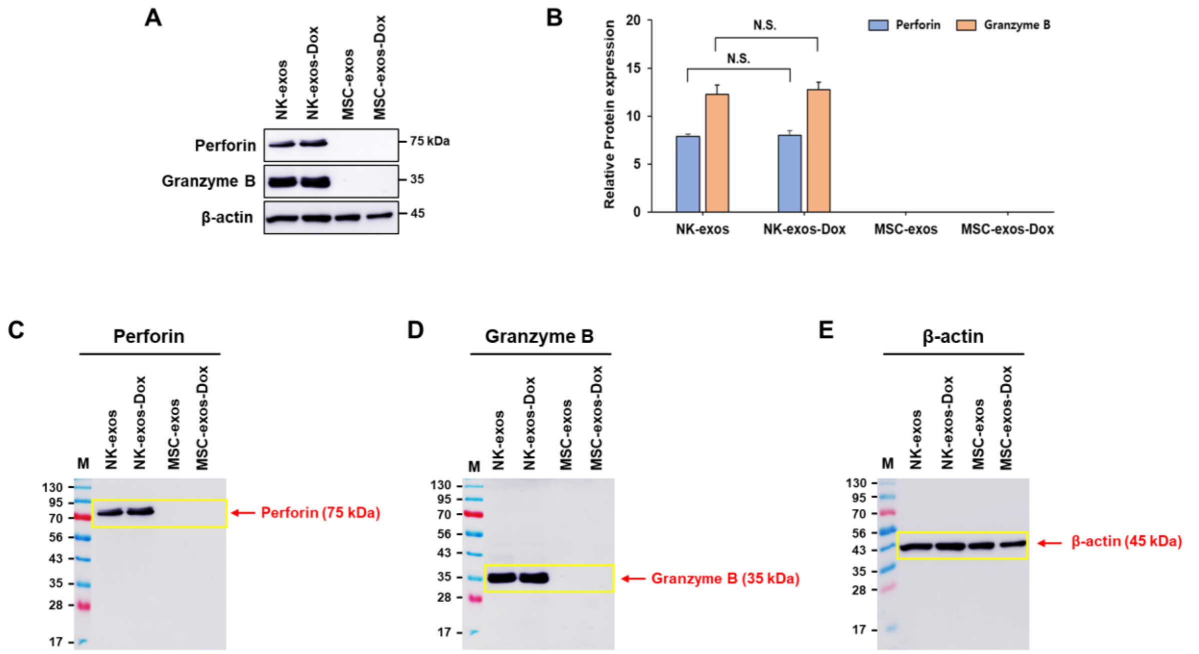

Figure S4. The expression levels of cytotoxic proteins in MSC-exos and MSC-exos-Dox as a negative control. **(A)** The cytotoxic protein expressions, including perforin and granzyme B were detected via western blot analysis. β-actin was used as a loading control. **(B)** Quantification of protein expression normalized to that of β-actin. All data are presented as the mean ± SD (n = 3). *N.S.* (no significance) vs. NK-exos. **(C-E)** Uncropped western blot images of perforin (C), granzyme B (D), and β-actin (E). Bands marked in yellow were used in Figure S4A. **Abbreviations**: MSC, mesenchymal stem cells; MSC-exos, mesenchymal stem cell-derived exosomes; MSC-exos-Dox, mesenchymal stem cell-derived exosomes loaded with doxorubicin.

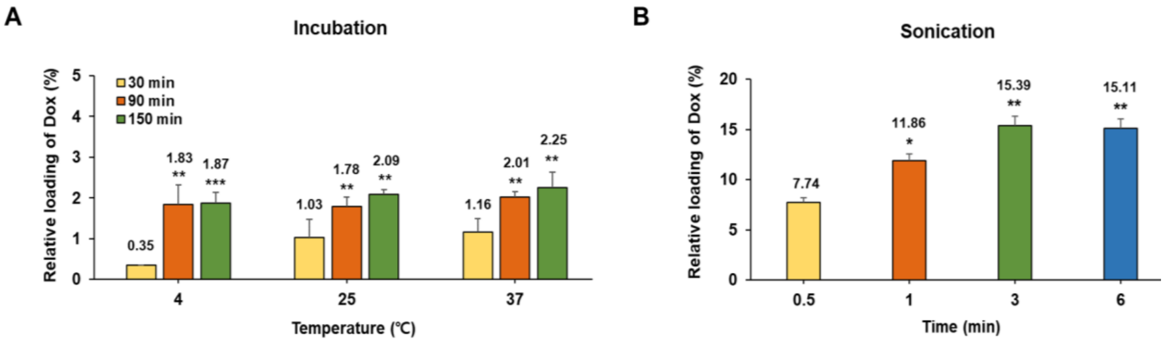

Figure S5. Comparison of the loading efficiency of Dox in conventional loading methods. **(A)** The relative loading efficiency of Dox was determined by incubating at 4, 25, and 37 ℃ for the specified durations. **(B)** The relative loading efficiency of Dox was evaluated using sonication for the indicated times. All data are presented as the mean ± SD ($n = 3$). $^{*}p < 0.05$, $^{**}p < 0.01$, and $^{***}p < 0.001$.

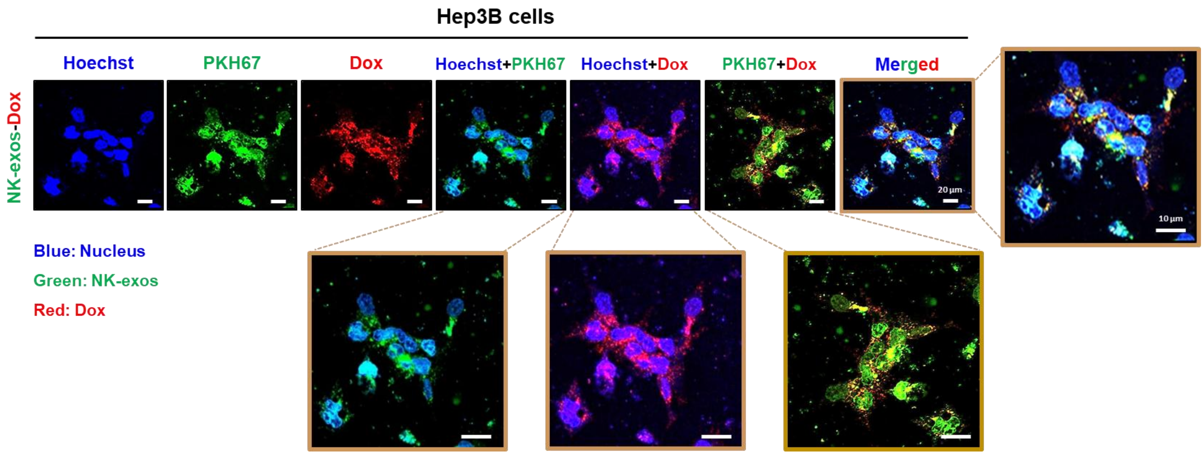

Figure S6. Cellular uptake of NK-exos-Dox by HCC cells from Figure 3A. Hep3B cells (1 × 10^4) were co-cultured with PKH67-labeled NK-exos-Dox (50 µg) for 24 h. Internalization was visualized by confocal microscopy. Free Dox, PKH67-labeled NK-exos, and cell nuclei were stained with red, green, and blue (Hoechst), respectively. Scale bars: 20 µm (left) and 10 µm (right).

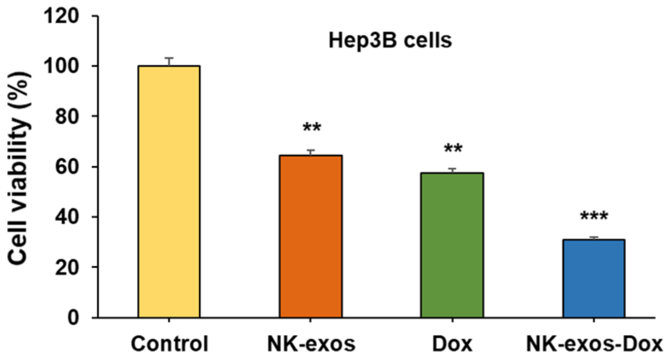

Figure S7. Cell viability effects of NK-exos-Dox in HCC cells using MTT assay. Hep3B cells (1×10^4) were co-treated with Dox (5 µg), NK-exos (50 µg), and NK-exos-Dox (50 µg) for 24 h. MTT solution (10 µL) was added to each well and re-incubated for 3 h. The medium was then removed, and DMSO (100 µL) was added. Cell viability was determined by measuring the absorbance at 570 nm using a microplate reader. Data are presented as the mean ± SD (n = 3). $^{**}p < 0.01$ and $^{***}p < 0.001$ vs. untreated Hep3B cells (control). **Abbreviations**: MTT, 3-(4,5-Dimethylthiazol-2-yl)-2,5-diphenyltetrazolium bromide; DMSO, dimethyl sulfoxide.

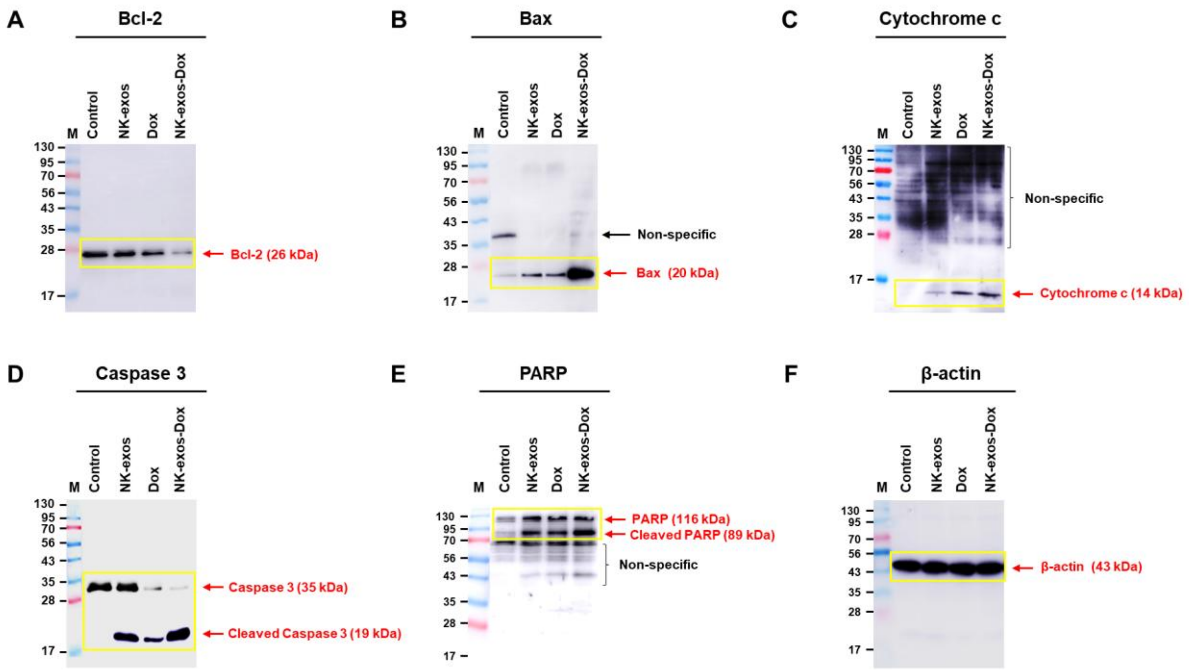

Figure S8. Uncropped western blots of anti-apoptotic and pro-apoptotic protein effects in Hep3B cells from Figure 5C. The related anti- or pro-apoptotic proteins were detected via western blot analysis. **(A)** The expression levels of anti-apoptotic protein, Bcl-2. **(B-E)** The expression levels of pro-apoptotic proteins, Bax (B), cytochrome c (C), caspase 3 (D), and PARP (E). **(F)** β-actin expression is a loading control marker. Specific expression band was indicated the yellow box and red arrow. **Abbreviations**: M, protein marker; Bcl-2, B-cell lymphoma 2; Bax, Bcl-2-associated X protein; Caspase 3, cysteine-aspartic acid protease 3; PARP, poly (ADP-ribose) polymerase.

Source	Target cancer	Target cell	Drug	Drug loading method	Biological effect			*In vitro* or *In vivo*	Ref
					Biomolecule	mechanisms	Functions		
NK-92	Breast cancer	MCF-7	Paclitaxel	Electroporation	Bax, Bcl-2<br>Caspase 3	Intrinsic apoptosis	Enhance apoptosis<br>Decline migration	*In vitro*	[1]
NK-92MI	Ovarian cancer	SKOV3<br>COC1	Cisplatin	Electroporation	Caspase 3, 7<br>PARP<br>CXCL 5, 8, 9, 10, 11	Intrinsic apoptosis	Enhance cytotoxicity<br>Enhance apoptosis	*In vitro*	[2]
NK-92	Liver cancer	Hep3B	Doxorubicin	Electroporation	Bax, Bcl-2<br>Caspase 3, PARP<br>Cytochrome c	Intrinsic apoptosis	Enhance cytotoxicity<br>Reduce cell viability<br>Enhance apoptosis	*In vitro*	In this study

Table S1. Biological functions and mechanisms of drug-loaded NK-exos. NK, natural killer; NK-92MI, interleukin-2-independent cell line derived from natural killer-92; MCF-7, michigan cancer foundation-7; Bcl-2, B-cell lymphoma 2; Bax, Bcl-2-associated X; Caspase 3, cysteine-aspartic acid protease 3; Caspase 7, cysteine-aspartic acid protease 7; PARP, Poly (ADP-ribose) polymerase; CXCL, C-X-C motif chemokine ligand; N/A, not applicable; Ref, reference.

Supplementary Table S1 References

[1] Han D, et al. Natural killer cell-derived exosome-entrapped paclitaxel can enhance its antitumor effect. Eur Rev Med Pharmacol Sci. 2020; 24(10): 5703-5713.

[2] Luo HY, et al. NK cell-derived exosomes enhance the anti-tumor effects against ovarian cancer by delivering cisplatin and reactivating NK cell functions. Front Immunol. 2023; 13: 1087689.
